# Validation of ^18^F-FDG PET/MRI and diffusion-weighted MRI for estimating the extent of peritoneal carcinomatosis in ovarian and endometrial cancer -a pilot study

**DOI:** 10.1186/s40644-021-00399-2

**Published:** 2021-04-13

**Authors:** Björg Jónsdóttir, Montserrat Alemany Ripoll, Antonina Bergman, Ilvars Silins, Inger Sundström Poromaa, Håkan Ahlström, Karin Stålberg

**Affiliations:** 1 Department of Women’s and Children’s Health, Uppsala University, 75185 Uppsala, Sweden; 2 Department of Surgical Sciences, Section of Radiology, Uppsala University, Uppsala, Sweden; 3 Antaros, Medical AB, Uppsala, Sweden

**Keywords:** PET/MRI, DW-MRI, Ovarian cancer, Peritoneal cancer index (PCI), Carcinomatosis

## Abstract

**Background:**

The extent of peritoneal carcinomatosis is difficult to estimate preoperatively, but a valid measure would be important in identifying operable patients**.**

The present study set out to validate the usefulness of integrated ^18^F-FDG PET/MRI, in comparison with diffusion-weighted MRI (DW-MRI), for estimation of the extent of peritoneal carcinomatosis in patients with gynaecological cancer.

**Methods:**

Whole-body PET/MRI was performed on 34 patients with presumed carcinomatosis of gynaecological origin, all scheduled for surgery. Two radiologists evaluated the peritoneal cancer index (PCI) on PET/MRI and DW-MRI scans in consensus. The surgeon estimated PCI intraoperatively, which was used as the gold standard.

**Results:**

Median total PCI for PET/MRI (21.5) was closer to surgical PCI (24.5) (*p* = 0.6), than DW-MRI (median PCI 20.0, *p* = 0.007). However, both methods were highly correlated with the surgical PCI (PET/MRI: β = 0.94 *p* < 0.01, DW-MRI: β = 0.86, *p* < 0.01). PET/MRI was more accurate (*p* = 0.3) than DW-MRI (*p* = 0.001) when evaluating patients at primary diagnosis but no difference was noted in patients treated with chemotherapy. PET/MRI was superior in evaluating high tumour burden in inoperable patients. In the small bowel regions, there was a tendency of higher sensitivity but lower specificity in PET/MRI compared to DW-MRI.

**Conclusions:**

Our results suggest that FDG PET/MRI is superior to DW-MRI in estimating total spread of carcinomatosis in gynaecological cancer. Further, the greatest advantage of PET/MRI seems to be in patients at primary diagnosis and with high tumour burden, which suggest that it could be a useful tool when deciding about operability in gynaecological cancer.

**Supplementary Information:**

The online version contains supplementary material available at 10.1186/s40644-021-00399-2.

## Introduction

Ovarian cancer is the fifth most common cause of cancer-related deaths in women in Europe with a five-year survival of around 40% [1]. The age-standardised incidence rate is around 12–14/100.000 in the Nordic countries [2], with 751 cases diagnosed in Sweden in 2013 [3]. Ovarian cancer usually presents at an advanced stage, with intra-abdominal spread and peritoneal carcinomatosis at diagnosis. The mainstay of treatment is primary surgery, aiming at complete cytoreduction, followed by chemotherapy [4, 5]. Because of the widespread disease, surgery is often extensive and not suitable for all patients. It is of great value to identify patients with non-resectable tumours who preferably should benefit from neoadjuvant chemotherapy. Endometrial cancer is more seldom diagnosed in advanced stage, but in these women, the prognosis and treatment is similar to advanced ovarian cancer [6].

Carcinomatosis can be estimated intraoperatively by using the peritoneal cancer index (PCI) [7], which describes the distribution of tumours in the abdomen and pelvis. PCI correlates with the completeness of cytoreduction, and is associated with long-term survival [8, 9]. Furthermore, it has been suggested that it can be used to estimate operability; however, the cut-off point is yet to be determined. PCI has also been used in radiology in an effort to obtain this information preoperatively.

There is no universally accepted reference standard for imaging peritoneal carcinomatosis. Prospective studies comparing CT, PET-CT and MRI are few, and findings indicate minor differences between the methods [10]. Contrast-enhanced CT is to date the imaging modality of choice but has limitations in detecting carcinomatosis and small bowel involvement [11, 12]. PET-CT has been shown to be sensitive and specific to the detection of lymph node and distant metastases, and is therefore mostly used in detecting recurrent disease and response monitoring [13, 14]. MRI with diffusion-weighted imaging (DWI) has also been suggested for preoperative staging of abdominal carcinomatosis and might be more accurate than CT [15]. Recently, MRI including DWI has shown superior results to CT in preoperative assessment of suspected ovarian cancer [16].

A combination of PET and MRI was recently introduced, which may have advantages over PET/CT [17] as MRI is superior for morphology and adds functional information in terms of DWI [18]. The few studies available suggest the high diagnostic potential of PET/MRI for the assessment of recurrence of female pelvic malignancies [18, 19], and higher diagnostic confidence in discrimination between benign and malignant lesions compared with PET/CT [19]. However, previous published studies are few and heterogeneous, and to the best of our knowledge, no previous studies evaluating carcinomatosis with PET/MRI have been published.

The purpose of this prospective study was to validate integrated PET/MRI, in comparison with DW-MRI alone, for preoperative prediction of peritoneal carcinomatosis with gynaecological origin.

## Material and methods

None of the authors declared any conflict of interest regarding the subject of this study. All patients were willing to participate in the study and signed written informed consent. The study procedures were in accordance with ethical standards for human experimentation and the study was approved by the local ethics committee.

### Patients

This is a prospective, non-randomized single institution study of patients treated at one centre. In the comparative analyses the study participants act as their own controls.

Women admitted to the Department of Gynaecology, Akademiska Sjukhuset, Uppsala for cytoreductive surgery were considered for the study. Inclusion criteria were suspected peritoneal carcinomatosis of gynaecological origin (ovarian, fallopian tube or endometrial cancer) where operation was considered possible. Preoperative diagnosis was based on cytology, histology, tumour markers, and CT scan of the abdomen and thorax. Exclusion criteria were clearly inoperable patients (such as those with extra-abdominal metastases on CT or poor medical conditions) or known contraindications for MRI, such as metal foreign bodies, pacemakers and claustrophobia. Patients were included at primary diagnosis or after successful treatment with neoadjuvant chemotherapy.

After providing written informed consent they were all enrolled for a whole-body PET/MRI scan in addition to previous diagnostic imaging. Clinical treatment decision was made primarily on the results from the CT scan, but occasionally findings were confirmed on PET/MRI. The results from the radiologic PCI-evaluation were not available preoperatively.

Patients entered the study from June 2015 to May 2018. However, the total number of patients was restricted due to logistic aspects such as long travelling distances and limited access to the PET/MRI camera within the close time frame from diagnosis to surgery.

#### Peritoneal Cancer index (PCI)

Peritoneal cancer index is a scoring system that combines lesion size with tumour distribution leading to a numeric score, which quantifies the extent of disease. For anatomical distribution 13 regions are defined. Two transverse planes and two sagittal planes divide the abdominopelvic cavity into nine regions, which are numbered in a clockwise direction with 0 at the umbilicus. Regions 9–12 divide the small bowel. Lesion size (LS) refers to the greatest diameter of tumour implants that are distributed on the peritoneal surfaces, ranging from LS 0 (no tumour seen) to LS 3 (tumour > 5 cm). If there is a confluence of disease matting abdominal or pelvic structures together, this is automatically scored as LS 3 even if it is a thin confluence of cancerous implants. Peritoneal cancer index, thus, ranges from 0 to 39. Primary tumours are excluded from the LS lesion size assessment [6] (Supplementary Table 1).

#### Radiology

After standard patient preparation, 2 MBq/kg of Fluorodeoxyglucose (^18^F-FDG) was injected intravenously 60 min prior to image acquisition. A whole-body PET/MRI was performed from the skull base to the proximal femur using an integrated whole-body PET/MR 3.0 T scanner (SIGNA PET/MR, GE Healthcare). All patients received intravenous contrast agent, gadoterate meglumine (Dotarem) 0.1 mmol/kg and 20 mg buscopan i.m. No peroral contrast was given. The MRI sequences were acquired simultaneously with the PET data and consisted of whole-body diffusion weighted imaging with background suppression (DWIBS), 2 b-factors (50, 1000) (TR 3800 ms, TE 63 ms, TI 246 ms, BW 250 kHz, Matrix 96 × 128, FOV 44x35cm, Slice/gap 6/0 mm, Scantime 2:27 min), and whole-body T1-w DIXON after i.v. contrast agent (TR 4,1 ms, TE 1,1/2,2 ms, Bandwith (BW) 166 kHz, Matrix 256 × 212, FOV 50x45cm, Slice/gap 5/− 2,5 mm, Scantime 17sek) and FSE T2-w images for anatomical correlation (TR 2000 ms, TE 102 ms, BW 62,5 kHz, Matri× 320 × 224, FOV 44x35cm, Slice/gap 6/0 mm, Scantime 43 s = Breath Hold (BH) × 3 (14,5sek/BH).

The images were evaluated by two radiologists with long experience in interpretation of both MRI and PET image (M.A. and H.A.), first separately and then in consensus in case of disagreement. The radiologists were blinded to the patients’ medical history and surgical findings. Two different radiologic PCI scores were calculated for each patient: one for MRI alone (MR images separated from PET/MRI), based on DWI, and one for fused PET/MRI simultaneously acquired images. A focal lesion located at any of the 13 abdominopelvic regions (S Table 1) was considered a peritoneal metastasis by MRI if it had restricted diffusion (high signal intensity on DWI b-factor 1000, with corresponding low signal intensity compared with surrounding normal tissue on apparent diffusion coefficient (ADC) map by visual analysis and, ideally, if it had a morphological correlation on T1- or at T2-w images. For a lesion to be considered a peritoneal metastasis by PET/MRI it had to show hyper-metabolic activity by PET (SUVmax > 4) or/and restricted diffusion by MRI. DWI was first analysed for all 34 included patients. The PET/MRI images were evaluated at least 1 month later in order to avoid bias.

**Table 1:** General characteristics of the study population

	Number of patients (%)
**Histology**	
Ovarian cancer:	31 (91.2)
High grade serous adenocarcinoma	26 (76.5)
Low grade serous adenocarcinoma	1 (2.9)
Clear cell carcinoma	1 (2.9)
Borderline ovarian tumor	2 (5.9)
Endometrioid adenocarcinoma	1 (2.9)
Endometrial cancer	3 (8.8)
Endometrioid adenocarcinoma	2 (5.9)
Serous adenocarcinoma	1 (2.9)
**FIGO stage**	
IB	1 (2.9)
IC	1 (2.9)
IIIB	1 (2.9)
IIIC	13 (38.2)
IV	18 (52.9)
**First treatment**	
Cytoreductive surgery	24 (70.6)
Neoadjuvant chemotherapy	10 (29.4)
**Ascites**	20 (58.8)

#### Surgery

Surgery was performed with the intention of removing all macroscopic tumours. Two surgeons specialized in gynaecological cancer surgery performed all surgeries and commenced the operation by evaluating PCI in a standardised manner. At the end of the surgery, the completeness of the cytoreduction score (CCS) [6] was estimated and recorded as follows: CC0: no residual disease; CC1: residual nodules measuring less than 2.5 mm; CC2: residual nodules measuring between 2.5 mm and 2.5 cm; and CC3: residual nodules greater than 2.5 cm. If the surgeon did not see any possibility for radical or near radical surgery (CC0-CC1), no surgery was performed and the abdomen was closed.

The radiologist and the surgeon were blinded to each other’s PCI scoring.

### Statistical analyses

The intraoperative PCI was considered the gold standard for the statistical analyses. The sensitivity, specificity and accuracy were calculated for each region, based on the presence versus absence of peritoneal implants regardless of size. The Bland-Altman method with regression analysis was used to calculate the bias of the DW-MRI and PET/MRI-derived peritoneal cancer index. In a secondary analysis we compared the total PCI and PCI for the small bowel regions, for PET/MRI and DW-MRI alone, in relation to first treatment (primary surgery vs NACT) and surgical outcome (macroscopic residual tumour) by Wilcoxon signed rank test. SPSS (Statistical Package for the Social Sciences) and Graph Pad software were used for the statistical analyses.

## Results

### Patients

In all, 37 women were included in the study. Three patients were excluded; one discontinued the examination due to claustrophobia, and two had cancers that were not of gynaecological origin (rectal cancer and smooth muscle tumour of uncertain malignant potential). The final study population consisted of a total of 34 patients, 31 with ovarian cancer and three with advanced endometrial cancer.

The mean age of the patients was 61 years (range 37–78, median 62). Twenty-six (76.5%) had high-grade serous adenocarcinoma. Thirty-one (91%) patients had stage IIIC or IV of the International Federation of Gynaecology and Obstetrics (FIGO) staging (Table 1). Twenty-four patients underwent primary surgery and ten patients were operated after neoadjuvant chemotherapy. The reasons for neoadjuvant chemotherapy were patient-related, such as low albumin level, high quantity of ascites or bad general health condition. PET/MRI was performed on average 8.6 days before surgery (range 1–29 days, median 6.0 days) and for those that had received neoadjuvant treatment PET/MRI was performed after the treatment and before surgery. At surgery, four patients had an inoperable tumour load, leading to maximum macroscopic residual tumour (CCS 3). The remaining 30 patients had complete cytoreductive surgery, with no macroscopic residual tumour (CCS 0).

### Peritoneal cancer index and surgical outcome

The total surgical PCI ranged from 3 to 37 (median 24.5), and corresponding ranges were 0–39 and 0–39 for DW-MRI and PET/MRI, respectively. The four patients with macroscopic residual tumour (inoperable) had the highest PCI surgical scores; 33–37.

**Fig. 1:**
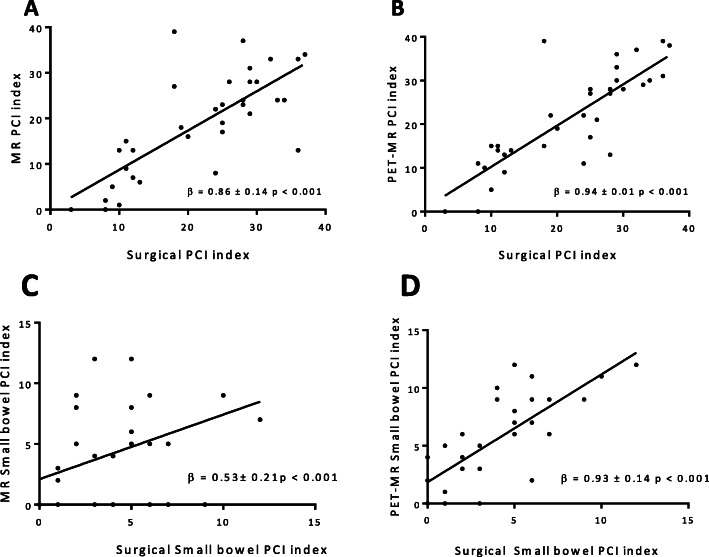
Correlations between surgical total PCI index and MRI PCI (**a**) and PET/MRI (**b**) and between surgical small bowel (region 9–12) PCI index and MRI small bowel PCI (**a**) and PET/MRI small bowel PCI index (**b**)

**Fig. 2:**
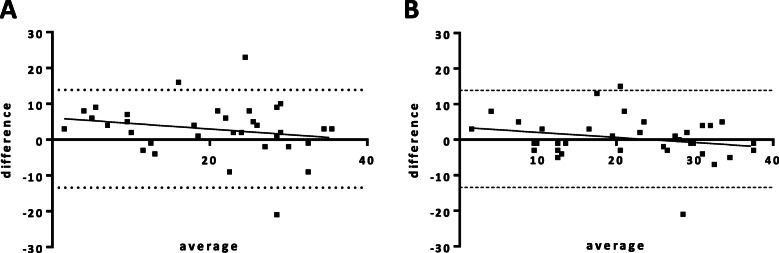
Bland-Altman plots comparing PCI index of DW-MRI (**a**) and PET/MRI (**b**) with surgical PCI index. DW-MRI bias was 2.91 ± 7.49, regression b = − 0.15 ± 0.13, *p* = 0.24. PET/MRI bias was 0.41 ± 6.24, regression b = − 0.14 ± 0.11, *p* = 0.19

Both DW-MRI- and PET/MRI-derived PCI were highly and positively correlated with the surgical PCI (DW-MRI: β = 0.86 ± 0.14 *p* < 0.01, PET/MRI: β = 0.94 ± 0.01 *p* < 0.01) (Fig. 1 a and b). In the Bland-Altman plot the mean difference of agreement (bias) for DW-MRI was slightly higher than for PET/MRI but the difference was not statistically significant (Fig. 2).

Specificity was higher for DW-MRI than PET/MRI in several regions, ranging for both methods from 50 to 100%. Accuracy was similar when comparing the two methods (Table 2).

**Table 2:** Sensitivity, specificity, and accuracy of PCI scores of PET/MRI and MRI with surgical PCI as gold standard

			DW-MRI PCI			PET/MRI PCI		
	PCI region	Patients with tumor found in surgery (n)	Sensitivity (%)	Specificity (%)	Accuracy (%)	Sensitivity (%)	Specificity (%)	Accuracy (%)
**PCI 0**	**Central**	28	71.4	66.7	73.5	82.1	50.0	76.4
**PCI 1**	**Right upper quadrant**	31	80.6	66.7	79.4	77.4	66.7	76.4
**PCI 2**	**Epigastrium**	24	68.3	80.0	64.7	58.3	80.0	64.7
**PCI 3**	**Left upper quadrant**	25	76.0	100.0	82.4	64.0	100.0	73.5
**PCI 4**	**Left flank**	23	82.6	72.7	79.4	73.9	63.6	70.6
**PCI 5**	**Left lower quadrant**	32	65.6	50.0	64.7	65.6	50.0	64.7
**PCI 6**	**Pelvis**	33	81.8	100	82.3	93.9	100	94.1
**PCI 7**	**Right lower quadrant**	31	61.3	100.0	64.7	67.7	100.0	70.6
**PCI 8**	**Right flank**	23	56.5	72.7	61.8	82.6	54.5	73.6
**PCI 9**	**Proximal jejunum**	15	46.7	68.4	58.8	73.3	57.9	64.7
**PCI 10**	**Distal jejunum**	18	55.6	66.7	58.8	72.2	87.5	79.4
**PCI 11**	**Proximal ileum**	23	52.2	100.0	67.6	43.5	81.8	55.9
**PCI 12**	**Distal ileum**	27	59.3	100.0	67.6	88.9	85.7	88.2

The median total PCI was lower for the ten patients who received neoadjuvant chemotherapy (15.5) than for patients who underwent primary surgery (27.0) (Table 3). The patient with largest discrepancy between surgical PCI and DW-MRI and PET/MRI (outlier in Fig. 1) had a surgical PCI of 18 but 39 in both DW-MRI and PET/MRI. This patient received neoadjuvant chemotherapy.

**Table 3:** Median total PCI scores and PCI scores for small bowel (region 9–12) from surgery, MRI and PET/MRI, patients grouped by surgical outcome and first treatment

	Total PCI Surgical (median, IQR)	Total PCI MRI (median, IQR)	p^**a**^	Total PCI PET/MRI (median, IQR)	p^**a**^	PCI Small bowel Surgical (median, IQR)	PCI Small bowel MRI (median, IQR)	p^**a**^	PCI Small bowel PET/MRI (median, IQR)	p^**a**^
**All patients** ***n*** **= 34**	24.5 (11.7–29.0)	20.0 (8.7–28.0)	0.007	21.5 (13.0–30.0)	0.6	3.5 (1.0–6.0)	4.0 (0.0–8.0)	0.531	5.5 (2.0–9.0)	0.001
**Primary surgery (*n*** **= 24)**	27 (13.5–31-5)	23 (8.5–28.0)	0.001	27.7 (12.0–30.8)	0.3	4.0 (1.3–6.0)	4.0 (0.0–8.0)	1.0	6.0 (2.3–9.0)	0.002
**NACT (*n*** **= 10)**	15.5 (11.0–24.3)	14.0 (8.8–25.8)	0.959	14.5 (13.0–23.3)	0.5	2.5 (1.0–5.0)	4.0 (0.0–7.59	0.26	4.5 (1.5–6.5)	0.139
**No macroscopic residual tumor** ***n*** **= 30**	22.0 (11.0–28.0)	18.5 (7.7–27.2)	0.031	18.0 (12.5–28..0)	0.8	3.0 (1.0–5.0)	4.0 (0.0–8.0)	0.074	5.0 (2.0–7.0)	0.002
**Macroscopic residual tumor (inoperable)** ***n*** **= 4**	36.0 (33.8–36.8)	28.5 (15.8–33.8)	0.066	34.5 (29.5–38.8)	0.5	9.5 (7.5–11.5)	3.5 (0.0–8.5)	0.068	10.0 (9.0–11.8)	0.2

^a^ In comparison with surgical PCI, Wilcoxon signed rank test. *IQR* interquartile range

The median total PCI obtained by PET/MRI (21.5, *p* = 0.6) did not differ significantly from the surgical PCI (24.5) whereas the DW-MRI-derived PCI was significantly lower (20.0, *p* = 0.007). Of the four patients considered inoperable, the median total surgical PCI was 36 and the corresponding score for PET/MRI was 34.5 (*p* = 0.5) and for DW-MRI 29 (*p* = 0.066) (Table 3).

### Regional peritoneal cancer index

PCI of the small bowel (region 9–12) was estimated separately (with a maximum score of 12). In these regions the correlation between DW-MRI and the surgical PCI was inferior (β = 0.53 ± 0.21 *p* < 0.01) than for PET/MRI: (β = 0.93 ± 0.14 *p* < 0.01) (Fig. 1b and c). In three out of four small bowel regions, PET/MRI displayed higher sensitivity for detection of carcinomatosis in comparison with DW-MRI (Table 2).

At primary surgery PET/MRI (median PCI 6.0) overestimated surgical small bowel PCI (median PCI 4.0, *p* = 0.002). For the 10 patients who received neoadjuvant chemotherapy the surgical PCI in the small bowel was 2.5 with scores 4.0 and 4.5 for DW-MRI and PET/MRI respectively (ns) (Table 3).

For the four patients with residual tumour after surgery a surgical PCI of 9.5 on the small bowel was found, with scores of 3.5 and 10 for DW-MRI and PET/MRI respectively (ns). For the patients with no macroscopic residual tumours PET/MRI significantly overestimated carcinomatosis on the small bowel (Table 3).

## Discussion

To the best of our knowledge, this is the first study estimating the extent of peritoneal carcinomatosis with integrated PET/MRI.

We found PET/MRI and DW-MRI-determined total peritoneal cancer index to be well correlated with the gold standard, surgical PCI. The PET/MRI PCI was significantly closer to the operative total PCI than DW-MRI alone. PET/MRI was more accurate than DW-MRI when evaluating patients at primary diagnosis but no difference was noted in patients previously treated with chemotherapy. PET/MRI was also superior in inoperable patients with high tumour burden, and in clinical practice, this is the most important finding, since these women could be offered neoadjuvant chemotherapy and not be subjected to unnecessary diagnostic laparotomy. In the small bowel regions, there was a tendency of higher sensitivity but lower specificity in PET/MRI compared to DW-MRI.

Few studies have evaluated PET/MRI in gynaecological cancer, and most of them focus on recurrent pelvic malignancies where PET/MRI may have advantages over PET-CT in better defining of lesion margins [17]. It has been shown that PET/MRI and PET-CT have equivalently high diagnostic value in recurrent cervical and ovarian cancer, but PET/MRI offers higher diagnostic confidence in the discrimination of benign and malignant lesions [19, 20]. Furthermore, a higher lesion contrast for malignant lesions has been found with PET/MRI than MRI alone [21]. In 2014, Schwenzer et al. [22] used PET/MRI to characterise carcinomatosis of different histology and found significant differences in glucose uptake and diffusion characteristics, depending on the histology of the tumours.

Peritoneal cancer index is a known prognostic indicator for operability of ovarian cancer [8]. Nevertheless, tumour load is not the only factor deciding surgical operability; the location of the tumour is also important. Massive small bowel infiltration is often the crucial factor that renders patients inoperable. It has previously been shown that the most difficult area for assessing carcinomatosis with radiology is the small bowel [12]. In our study, the sensitivity for carcinomatosis in the small bowel was higher for PET/MRI than for DW-MRI alone. One of the possible reasons is that the fusion of morphological, functional and metabolic information is highly accurate when acquired simultaneously in a hybrid PET/MRI system. However, PET/MRI overestimated peritoneal carcinomatosis in some bowel regions. This can be explained by the fact that bowels may appear hyper-intense in DWI due to long T2 relaxation times, impeded water movement, or both [23]. In addition, it is well known that FDG uptake in the gastrointestinal tract varies between individuals [24] with varying degrees of FDG uptake, usually with a diffuse and linear pattern but also with a focal pattern [25]. The mechanism of increased FDG uptake in the GI tract is unclear. It can be related to physiological activity in the bowel, to metabolically active smooth muscle, to reactive lymphocytes in the terminal ileum and cecum, or to swallowed secretions. These benign or physiological activities can be misinterpreted as malignant lesions and vice versa [26]. In addition, neoadjuvant therapy may induce increased FDG uptake because of inflammation simulating residual tumour.

In our clinical experience small bowel involvement is almost always associated with high overall tumour burden, consequently a low-moderate PCI and isolated small bowel involvement is rare. This is illustrated in this study where all four inoperable patients had a total PCI score > 33. In a recently published study by our group, total PCI was found an excellent predictor of incomplete cytoreductive surgery [27] hence we suggest that the radiologic evaluation could focus on estimating total tumour burden instead of separate tumour implants such as small bowel infiltration. This is in line with the findings in the current study with excellent correlation between PET/MRI and surgical evaluation especially when it comes to patients with high tumour burden who were considered inoperable.

**Fig. 3:**
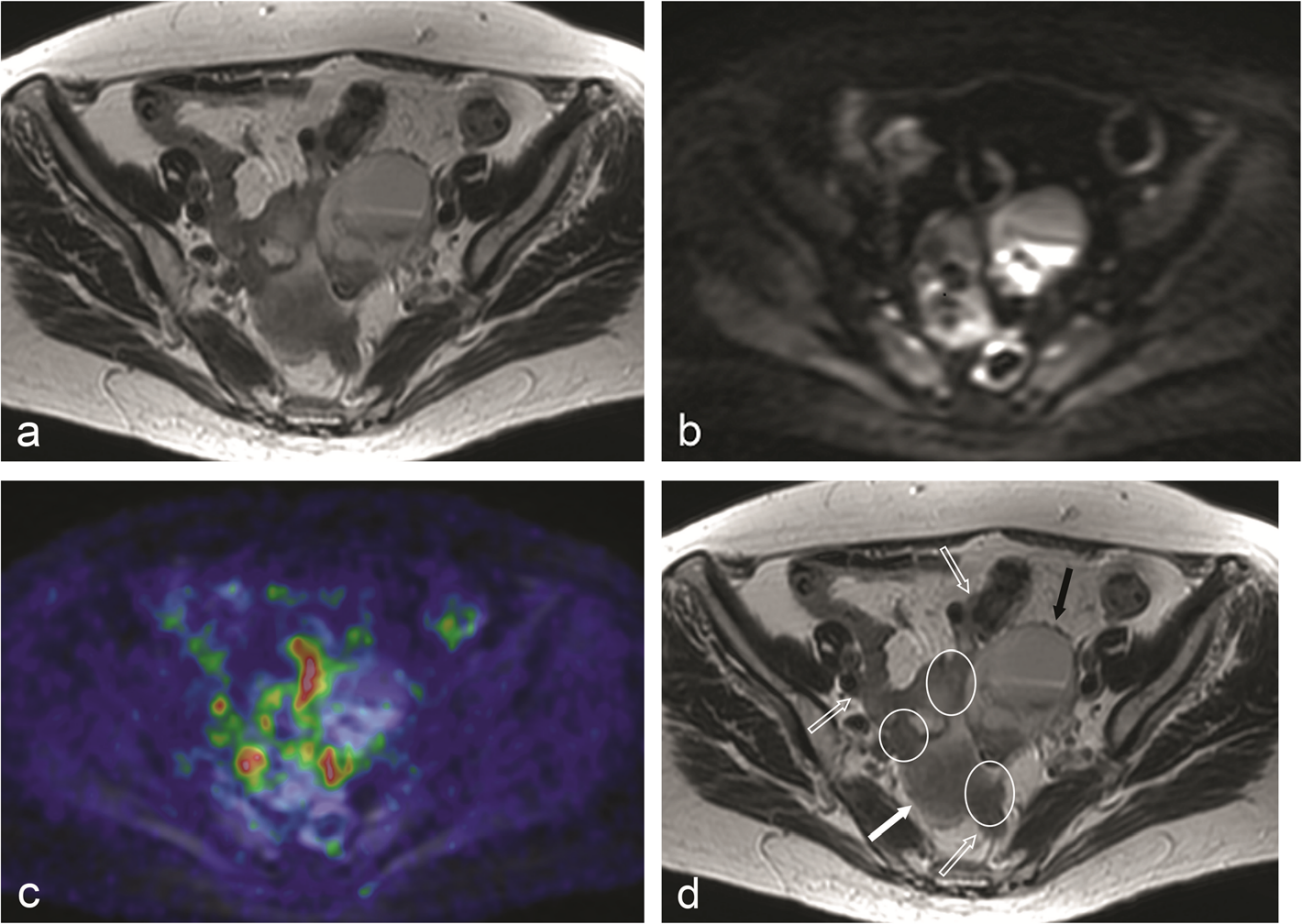
This FSE T2-w image (**a**) illustrates the difficulty in discriminating the different structures that contribute to form the complex image seen in the center of the pelvis. There are areas of restricted diffusion in DWI b = 1000 (**b**) which do not correspond to areas of increased FDG uptake as showed in this fused PET/MR image (**c**). The image was interpreted as being formed by a cystic ovarian tumor (solid black arrow), bowel loops (open arrows), peritoneal carcinomatosis (encircled) and fundus uteri (solid white arrow) (**d**). The exact localization of peritoneal implants, if they were on bowel surface or on the surface of pelvic peritoneum, was very difficult, as was to say if the loops corresponded to ileum or sigmoid colon. PCI for “Pelvis”, or “region 7”, was 3 for surgery, 0 for MRI alone (DWI) and 3 for PET/MR, thus a false negative for MRI and true positive for PET/MRI

**Fig. 4:**
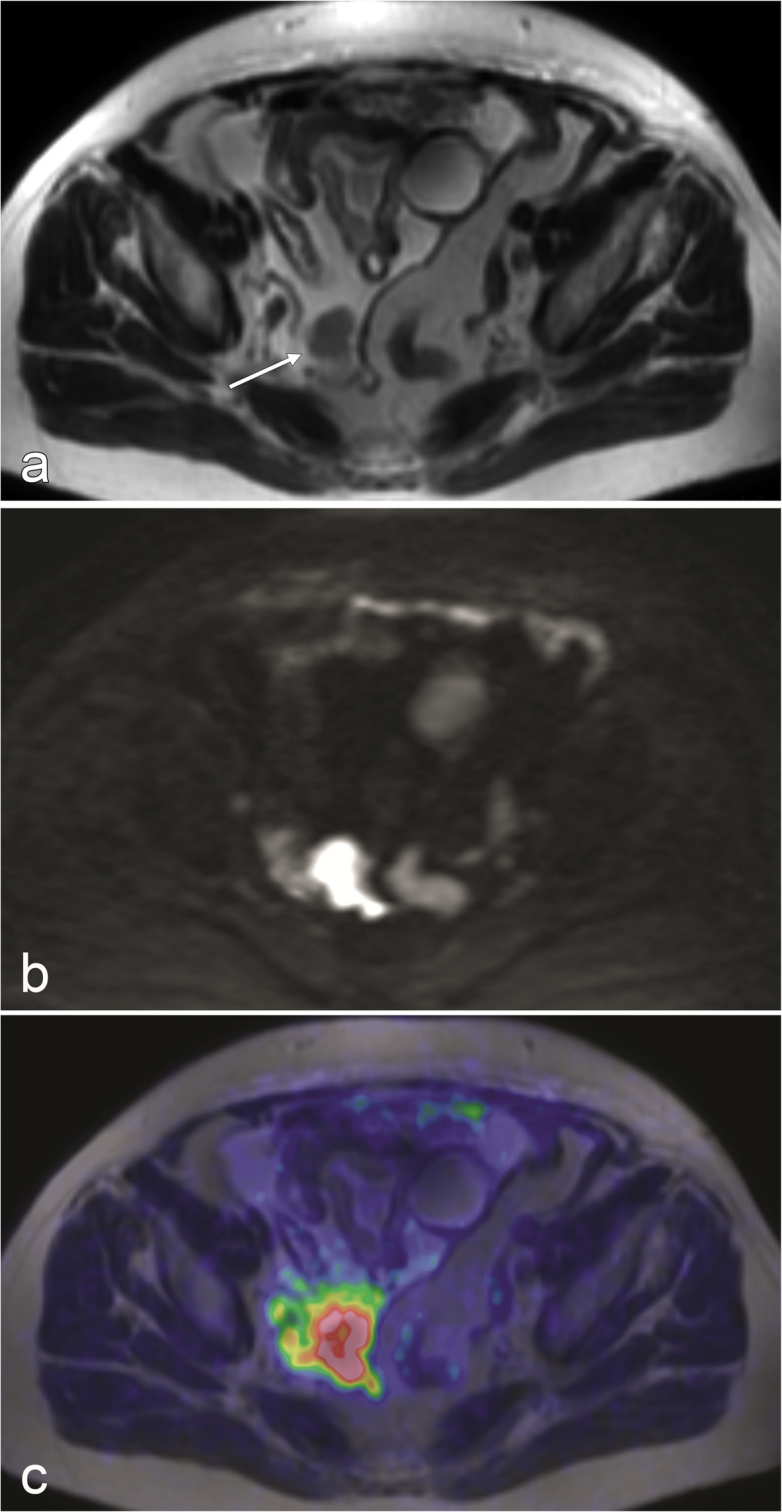
**a** FSE T2-w image: Ascites helped to correctly localize a peritoneal implant on the surface of pelvic peritoneum (arrow) by displacing adjacent bowel loops. The lesion was easily diagnosed either by MRI alone or by PET/MRI because of clear restriction of diffusion in DWI (**b**) and marked FDG uptake (**c**)

With both PET/MRI and DW-MRI, tumour burden in the individual regions differed from surgery. For example, peritoneal lesions assigned to region 5, the location of the sigmoid colon, were actually localised in the adjacent anatomic region or “lower jejunum”, and vice versa. These discrepancies might be explained by the fact that the surgeon moves organs to exactly define the location of peritoneal implants, but the radiologist interprets static images (Fig. 3). Nevertheless, the total PCI scores ended up similar, indicating that the tumour burden was equally well visualised even if its exact location was differently estimated. Two other common problems that led to mislocalisation were displacement of abdominal structures by large ovarian tumours and excessive amounts of ascites. However, sometimes ascites helped to correctly localise peritoneal lesions by separating otherwise adjacent structures (Fig. 4).

In the ten patients that had received neoadjuvant chemotherapy before radiology, we could not see any difference between the two methods, so presumably the additional advantage of PET is erased when the tumour activity is suppressed.

The study has its limitations, most importantly the relatively few patients and with the inclusion of both patients who had received neoadjuvant chemotherapy and those primarily operated on. Its strength lies in patients all having carcinomatosis of gynaecological origin, the majority being in the late FIGO stage. The same two surgeons assessed all patients during surgery in a standardised manner and two very experienced radiologists interpreted the images, further blinded to the patients’ medical history and surgical findings. Further, information on DWI-MRI and PET/MRI are gathered from the same individuals at the same time (with one examination) making patients their own controls and the examinations comparable.

## Conclusion

In the present study PET/MRI was superior to DW-MRI in estimating total peritoneal cancer index especially in patients with high tumour burden without previous chemotherapy. We further suggest that the radiologic evaluation could focus on estimating the total tumour burden instead of separate tumour implants, as it has an excellent correlation with operability. The exact role of PET/MRI in carcinomatosis of gynaecological origin cannot be defined by our study alone but our results are promising and justify similar studies with larger cohorts. We believe that PET/MRI could be a useful tool to help physicians when deciding about operability in ovarian cancer patients.

## Supplementary Information

**Additional file 1.**

## Data Availability

The datasets generated during the current study are not publicly available but are available from the corresponding author on reasonable request. However, HA is one of the founders of and employed by Antaros Medical AB. This company has not been involved in the presented study. Over the past 5 years, S-P has served occasionally on advisory boards or acted as invited speaker at scientific meetings for Asarina Pharma, MSD, Bayer Health Care, Gedeon Richter, Peptonics, Shire/Takeda, and Lundbeck A/S, these companies have not been involved in the presented study.

## Abbreviations

FDG: Fluorodeoxyglucose
PET/MRI: Positron emission tomography/magnetic resonance imaging
DW-MRI: Diffusion weighted-magnetic resonance imaging
PCI: Peritoneal cancer index
NACT: Neoadjuvant chemotherapy

## References

[1] Sant M, Chirlaque Lopez MD, Agresti R, Sanchez Perez MJ, Holleczek B, Bielska-Lasota M (2015). Survival of women with cancers of breast and genital organs in Europe 1999-2007: results of the EUROCARE-5 study. Eur J Cancer.

[2] Klint A, Tryggvadottir L, Bray F, Gislum M, Hakulinen T, Storm HH (2010). Trends in the survival of patients diagnosed with cancer in female genital organs in the Nordic countries 1964-2003 followed up to the end of 2006. Acta Oncol.

[3] Socialstyrelsen. Cancerincidens i sverige 2013 [Available from: http://www.socialstyrelsen.se/Lists/Artikelkatalog/Attachments/19613/2014-12-10.pdf. Accessed 4 Dec 2020.

[4] Bristow RE, Tomacruz RS, Armstrong DK, Trimble EL, Montz FJ (2002). Survival effect of maximal cytoreductive surgery for advanced ovarian carcinoma during the platinum era: a meta-analysis. J Clin Oncol.

[5] Luyckx M, Leblanc E, Filleron T, Morice P, Darai E, Classe JM, Ferron G, Stoeckle E, Pomel C, Vinet B, Chereau E, Bergzoll C, Querleu D (2012). Maximal cytoreduction in patients with FIGO stage IIIC to stage IV ovarian, fallopian, and peritoneal cancer in day-to-day practice: a retrospective French multicentric study. Int J Gynecol Cancer.

[6] Barlin JN, Puri I, Bristow RE (2010). Cytoreductive surgery for advanced or recurrent endometrial cancer: a meta-analysis. Gynecol Oncol.

[7] Jacquet P, Sugarbaker PH, Sugarbaker PH (1996). Clinical research methodologies in diagnosis and staging of patients with peritoneal carcinomatosis. Peritoneal Carcinomatosis: principles of management.

[8] Tentes AAK, Tripsiannis G, Markakidis SK, Karanikiotis CN, Tzegas G, Georgiadis G, Avgidou K (2003). Peritoneal cancer index: a prognostic indicator of survival in advanced ovarian cancer. Eur J Surg Oncol.

[9] Lampe B, Kroll N, Piso P, Forner DM, Mallmann P (2015). Prognostic significance of Sugarbaker's peritoneal cancer index for the operability of ovarian carcinoma. Int J Gynecol Cancer.

[10] Schmidt S, Meuli R, Achtari C, Prior J (2015). Peritoneal Carcinomatosis in primary ovarian Cancer staging comparison between MDCT, MRI, and 18F-FDG PET/CT. Clin Nucl Med.

[11] Suidan RS, Ramirez PT, Sarasohn DM, Teitcher JB, Mironov S, Iyer RB, Zhou Q, Iasonos A, Paul H, Hosaka M, Aghajanian CA, Leitao MM, Gardner GJ, Abu-Rustum NR, Sonoda Y, Levine DA, Hricak H, Chi DS (2014). A multicenter prospective trial evaluating the ability of preoperative computed tomography scan and serum CA-125 to predict suboptimal cytoreduction at primary debulking surgery for advanced ovarian, fallopian tube, and peritoneal cancer. Gynecol Oncol.

[12] Mazzei MA, Khader L, Cirigliano A, Cioffi Squitieri N, Guerrini S, Forzoni B, Marrelli D, Roviello F, Mazzei FG, Volterrani L (2013). Accuracy of MDCT in the preoperative definition of peritoneal Cancer index (PCI) in patients with advanced ovarian cancer who underwent peritonectomy and hyperthermic intraperitoneal chemotherapy (HIPEC). Abdom Imaging.

[13] Takekuma M, Maeda M, Ozawa T, Yasumi K, Torizuka T (2005). Positron emission tomography with 18F-fluoro-2-deoxyglucose for the detection of recurrent ovarian cancer. Int J Clin Oncol.

[14] Xu B, Ma J, Jiang G, Wang Y, Ma Q (2017). Diagnostic value of positron emission tomography (PET) and PET/computed tomography in recurrent/metastatic ovarian cancer: a meta-analysis. J Obstet Gynaecol Res.

[15] Low RN, Barone RM, Lucero J (2015). Comparison of MRI and CT for predicting the peritoneal Cancer index (PCI) preoperatively in patients being considered for cytoreductive surgical procedures. Ann Surg Oncol.

[16] Michielsen K, Dresen R, Vanslembrouck R, De Keyzer F, Amant F, Mussen E (2017). Diagnostic value of whole body diffusion-weighted MRI compared to computed tomography for pre-operative assessment of patients suspected for ovarian cancer. Eur J Cancer.

[17] Rosenkrantz AB, Friedman K, Chandarana H, Melsaether A, Moy L, Ding YS, Jhaveri K, Beltran L, Jain R (2016). Current status of hybrid PET/MRI in oncologic imaging. AJR Am J Roentgenol.

[18] Bailey DL, Antoch G, Bartenstein P, Barthel H, Beer AJ, Bisdas S, Bluemke DA, Boellaard R, Claussen CD, Franzius C, Hacker M, Hricak H, la Fougère C, Gückel B, Nekolla SG, Pichler BJ, Purz S, Quick HH, Sabri O, Sattler B, Schäfer J, Schmidt H, van den Hoff J, Voss S, Weber W, Wehrl HF, Beyer T (2015). Combined PET/MR: the real work has just started. Summary report of the third international workshop on PET/MR imaging; February 17-21, 2014, Tubingen, Germany. Mol Imaging Biol.

[19] Beiderwellen K, Grueneisen J, Ruhlmann V, Buderath P, Aktas B, Heusch P, Kraff O, Forsting M, Lauenstein TC, Umutlu L (2015). [(18) F] FDG PET/MRI vs. PET/CT for whole-body staging in patients with recurrent malignancies of the female pelvis: initial results. Eur J Nucl Med Mol Imaging.

[20] Grueneisen J, Schaarschmidt BM, Beiderwellen K, Schulze-Hagen A, Heubner M, Kinner S, Forsting M, Lauenstein T, Ruhlmann V, Umutlu L (2014). Diagnostic value of diffusion-weighted imaging in simultaneous 18F-FDG PET/MR imaging for whole-body staging of women with pelvic malignancies. J Nucl Med.

[21] Grueneisen JBK, Heusch P, Gratz M, Schulze-Hagen A, Heubner M (2014). Simultaneous positron emission tomography/magnetic resonance imaging for whole-body staging in patients with recurrent gynecological malignancies of the pelvis. A comparison to whole-body magnetic resonance imaging alone. Investig Radiol.

[22] Schwenzer NF, Schmidt H, Gatidis S, Brendle C, Muller M, Konigsrainer I (2014). Measurement of apparent diffusion coefficient with simultaneous MR/positron emission tomography in patients with peritoneal carcinomatosis: comparison with 18F-FDG-PET. J Magn Reson Imaging.

[23] Padhani AR, Koh DM, Collins DJ (2011). Whole-body diffusion-weighted MR imaging in cancer: current status and research directions. Radiology..

[24] Rosenbaum SJ, Lind T, Antoch G, Bockisch A (2006). False-positive FDG PET uptake--the role of PET/CT. Eur Radiol.

[25] Kostakoglu L, Agress H, Goldsmith SJ (2003). Clinical role of FDG PET in evaluation of cancer patients. Radiographics.

[26] Pandit-Taskar N, Schöder H, Gonen M, Larson SM, Yeung HW (2004). Clinical significance of unexplained abnormal focal FDG uptake in the abdomen during whole-body PET. AJR Am J Roentgenol.

[27] Jónsdóttir B, Lomnytska M, Poromaa IS, Silins I, Stålberg K. The Peritoneal Cancer Index is a Strong Predictor of Incomplete Cytoreductive Surgery in Ovarian Cancer. Ann Surg Oncol. 2021;28(1):244–51. 10.1245/s10434-020-08649-6. Epub 2020 May 29. 10.1245/s10434-020-08649-6 PMC7752870 32472412

