# Supplementary material for: Validation of 18F-FDG PET/MRI and diffusion-weighted MRI for estimating the extent of peritoneal carcinomatosis in ovarian and endometrial cancer -a pilot study
Source: Cancer Imaging. 2021 Apr 13;21:34. doi: 10.1186/s40644-021-00399-2 (PMC8042953; doi:10.1186/s40644-021-00399-2)
Supplement: Supplementary file 1 — Additional file 1. [file 40644_2021_399_MOESM1_ESM.docx]

**Appendix/supplementary data:**

**Supplementary Table 1:** Anatomic structures involved in 0-13 abdominopelvic regions of the peritoneal cancer index (PCI).

| Region 0 | Central midline abdominal incision | -Entire greater omentum, transverse colon |
| --- | --- | --- |
| Region 1 | Right upper | -Superior surface of the right lobe of the liver,  -Undersurface of the right hemidiaphragm,  -Right retro hepatic space |
| Region 2 | Epigastrium | -Epigastric fat pad,  -Left lobe of the liver and falciform ligament  -Lesser omentum, |
| Region 3 | Left upper | -Undersurface of the left hemidiaphragm,  -Spleen, tail of pancreas,  -Anterior and posterior surfaces of the stomach |
| Region 4 | Left flank | -Descending colon,  -Left abdominal gutter |
| Region 5 | Left lower | -Pelvic sidewall lateral to the sigmoid colon,  -Sigmoid colon |
| Region 6 | Pelvis | -Female internal genitalia with ovaries, tubes and uterus, -Douglas pouch,  -Bladder and rectosigmoid colon |
| Region 7 | Right lower | -Right pelvic sidewall,  -Cecum,  -Appendix |
| Region 8 | Right flank | -Right abdominal gutter,  -Ascending colon |
| Region 9 | Proximal jejunum |  |
| Region 10 | Distal jejunum |  |
| Region 11 | Proximal ileum |  |
| Region 12 | Distal ileum |  |
